# Peplomer bulb shape and coronavirus rotational diffusivity

**DOI:** 10.1063/5.0048626

**Published:** 2021-03-30

**Authors:** M. A. Kanso, V. Chaurasia, E. Fried, A. J. Giacomin

**Affiliations:** 1 Chemical Engineering Department, Polymers Research Group, Queen's University, Kingston, Ontario K7L 3N6, Canada; 2 Okinawa Institute of Science and Technology, Tancha, Onna, Kunigami District, Okinawa 904-0495, Japan; 3 Mechanical and Materials Engineering Department, Queen's University, Kingston, Ontario K7L 3N6, Canada; 4 Physics, Engineering Physics and Astronomy Department, Queen's University, Kingston, Ontario K7L 3N6, Canada

## Abstract

Recently, the rotational diffusivity of the coronavirus particle in suspension was calculated, from first principles, using general rigid bead-rod theory [M. A. Kanso, Phys. Fluids **32**, 113101 (2020)]. We did so by beading the capsid and then also by replacing each of its bulbous spikes with a single bead. However, each coronavirus spike is a glycoprotein trimer, and each spike bulb is triangular. In this work, we replace each bulbous coronavirus spike with a bead triplet, where each bead of the triplet is charged identically. This paper, thus, explores the role of bulb triangularity on the rotational diffusivity, an effect not previously considered. We thus use energy minimization for the spreading of triangular bulbs over the spherical capsid. The latter both translates and twists the coronavirus spikes relative to one another, and we then next arrive at the rotational diffusivity of the coronavirus particle in suspension, from first principles. We learn that the triangularity of the coronavirus spike bulb decreases its rotational diffusivity. For a typical peplomer population of 74, bulb triangularity decreases the rotational diffusivity by 39%.

## I. INTRODUCTION

**FIG 1.**
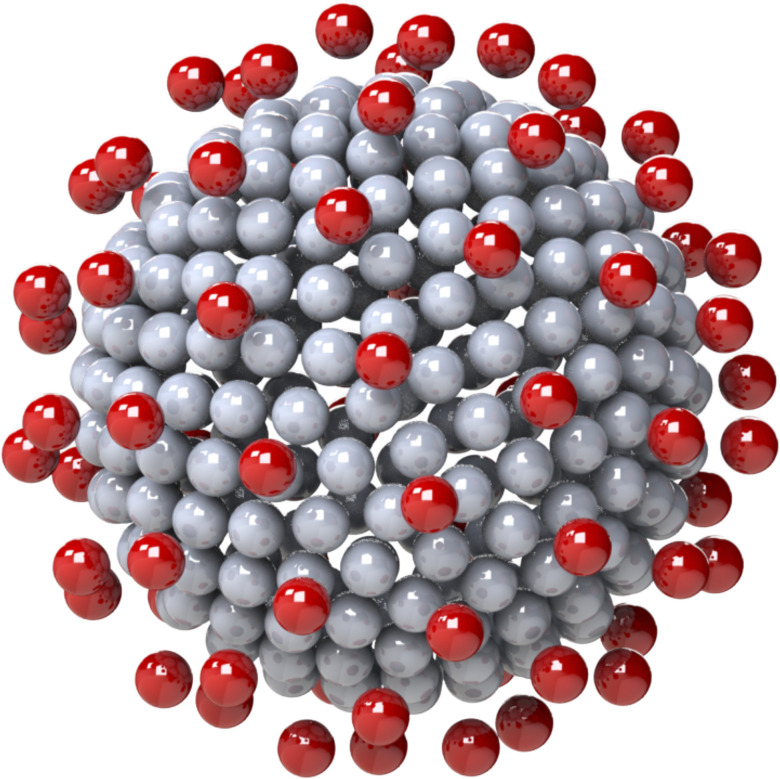
General rigid bead-rod model of single beaded coronavirus, $N_{c}=256$, $N_{p}=74$ (row 1 of Table III).

Recently, we calculated the rotational diffusivity of the coronavirus particle in suspension as a function of peplomer population, from first principles, using general rigid bead-rod theory (Fig. 12 of Ref. 1). We did so by beading the capsid and then also by replacing each of its bulbous spikes with a single bead (Fig. 1). One of the challenges of *ab initio* calculations from general rigid bead-rod theory on coronaviruses is that the peplomer arrangement is not known. However, we do know that the spikes are charge-rich.^2,3^ It also seems reasonable to assume that they are charged identically. Furthermore, we know that the coronavirus spikes are not anchored into its hard capsid, but rather just into its elastic viral membrane (Sec. 1 of Ref. 4). The coronavirus spikes are, thus, free to rearrange under their own electrostatic repulsions. This is why coronavirus spikes normally present microscopically as uniformly distributed over the capsid. In our previous work, we followed the well-known polyhedral solutions to the Thomson problem for singly charged particles repelling one another over a spherical surface.^5–7^ By *Thomson problem*, we mean determination of how identically charged particles repel and then spread over a sphere by minimizing system potential energy. This minimum system electrostatic potential energy, when divided by the sphere area, is not to be confused with surface energy.

**FIG. 2.**
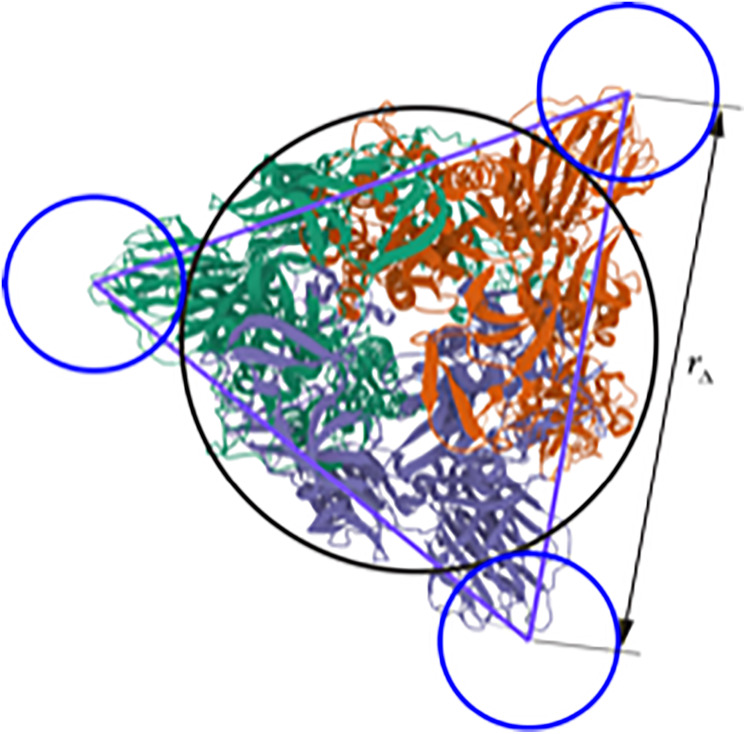
This paper explores the role of bulb triangularity (three blue circles) on the rotational diffusivity. This improves upon the previously considered singly beaded bulb (black circle).^1^ Bulb-end view of trimeric SARS-CoV peplomer (generated using 6CRZ.PDB data available from Ref. 15).

**FIG. 3.**
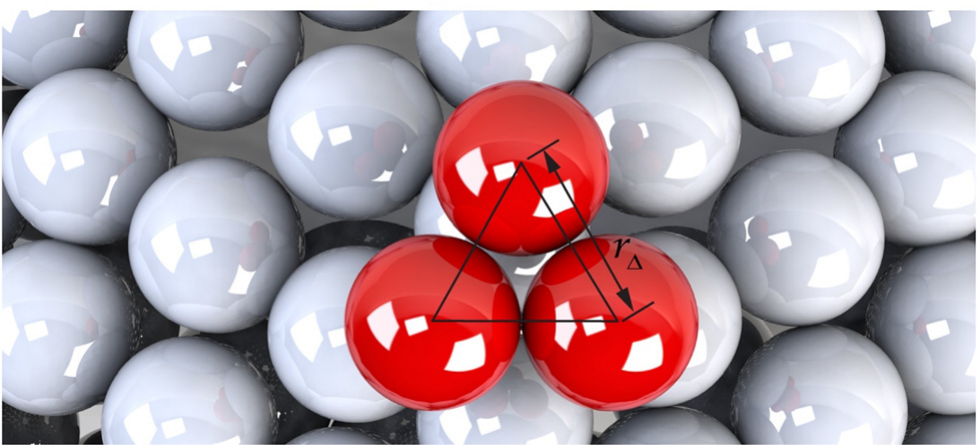
General rigid bead-rod model of three beads peplomer head.

Since each coronavirus spike is a glycoprotein trimer, each spike bulb is triangular (Fig. 14 of Ref. 1). By replacing each coronavirus spike bulb with a single bead (see circle in Fig. 14 of Ref. 1), our prior work neglects this triangularity. In this present work, we replace each bulbous coronavirus spike (Fig. 2) with a bead triplet (Fig. 3), with each bead identical and charged identically. We must, thus, replace the well-known polyhedral solutions to the single-bead Thomson problem with our new solutions to the triple-bead Thomson problem. In this work, we thus use minimum potential energy peplomer arrangements for our coronavirus model particles.

**FIG. 4.**
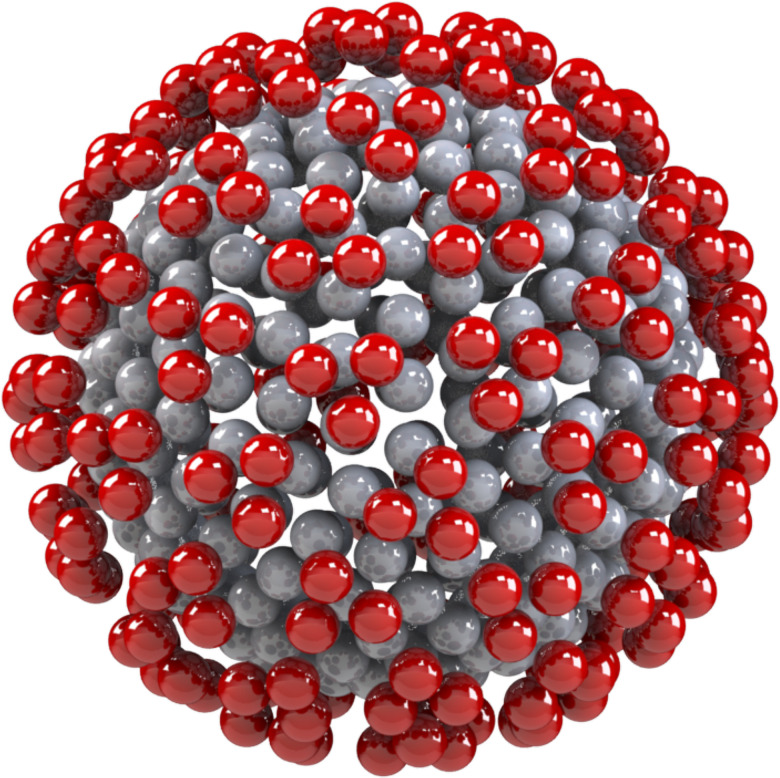
General rigid bead-rod model of triple beaded coronavirus, $N_{c}=256$, $N_{p}=74$ (row 2 of Table III).

Since coronavirus bulbs are trimers, they not only translate into a set of centroidal positions relative to one another but also twist into a set of orientations relative to one another. Our potential energy minimization for our triply beaded peplomers thus yields both triplet positions and triplet orientations (Fig. 4). This new potential energy minimization yields a set of bead positions for the triply beaded peplomers whose centroid positions differ, of course, from the bead positions for the singly beaded counterpart of the same $N_{p}.$ In other words, the polyhedra of centroids differ from the well-known Thomson solutions used in Ref. 1.

The challenge in determining the rotational diffusivity of a virus particle, from first principles, begins with modeling its intricate geometry with beads, locating the position of each bead. Once overcome, the next challenge is to use this geometry to arrive at the transport properties for the SARS-CoV-2 particle. From these, we deepen our understanding of how these remarkable particles align their peplomers both for long enough, and often enough, to infect.^1^

Whereas our prior work relied on the Thomson solution for point charges (Fig. 1), here, we work with triads of point charges each spaced rigidly and equilaterally (Fig. 4). We, thus, complicate the energy minimization with the length of this equilateral triangle, $r_{\Delta }$. From Table X of Ref. 1, mindful of Fig. 8 of Ref. 1, we get

$$\frac{1}{10}\leq \frac{r_{\Delta }}{r_{p}}\leq \frac{7}{25},$$ (1)

and in this work, we choose $r_{\Delta }/r_{p}=0.19$ for our energy minimization. To compare with our previous work, we match the dimensionless virus radius of Fig. 12 of Ref. 1, $r_{v}/r_{c}=5/4$. Using the energy minimization to arrange and orient the coronavirus spikes relative to one another, we next arrive, from first principles, at the rotational diffusivity of the coronavirus particles with triple beaded peplomers in suspension.

**TABLE I.** Dimensional variables.

Name	Unit	Symbol
Angular frequency	$t^{-1}$	ω
Bead diameter	L	d
Capsid radius	L	$r_{c}$
Complex viscosity	M/Lt	$\eta^{*}$
Dielectric permittivity	$T^{4}I^{2}/ML^{3}$	*ϵ*
Energy values in molecular-scale systems	$ML^{2}/t^{2}$	kT
Length of the equilateral triangle forming bead centers of triadic bulb	L	$r_{\Delta }$
Length of the spike of each peplomer	L	ℓ
Minus imaginary part of non-linear complex viscosity	M/Lt	$\eta^{\prime \prime }$
Moments of inertia	$ML^{2}$	$I_{1},I_{2},I_{3}$
Number of dumbbells per unit volume	$1/L^{3}$	n
Peplomer bulb center radial position	L	$r_{p}\equiv r_{v}-r_{b}$
Peplomer sphere radius, s=1,2,3	L	$r_{j,s}$
Peplomer vertex radius, q=1,2,3	L	$r_{i,q}$
Point charge	A s	Q
Real part of non-linear complex viscosity	M/Lt	$\eta^{\prime }$
Relaxation time of rigid dumbbell	t	$\lambda_{0}$
Relaxation time of solution	t	λ
Rotational diffusivity	$s^{-1}$	$D_{r}$
Shear rate amplitude	$t^{-1}$	${\dot{\gamma }}^{0}$
Solvent viscosity	M/Lt	$\eta_{s}$
Sphere radius	L	$r_{s}=r_{c}+\ell$
Temperature	T	T
Time	t	t
Total electrostatic energy	$ML^{2}/t^{2}$	E
Virus radius	L	$r_{v}$
Viscosity, zero-shear	M/Lt	$\eta_{0}$
Zero-shear first normal stress difference	M/L	$\Psi_{1,0}$

Legend: *M* ≡ mass; *L* ≡ length; *t* ≡ time.

## II. METHOD

For this work, we chose general rigid bead-rod theory for its flexibility and accuracy (Sec. I of Refs. 8 and 9). Using general rigid bead-rod theory, we follow the method of Sec. II of Ref. 1 to construct our virus particles from sets of beads whose positions are fixed relative to one another. For example, the SARS-CoV-2 particle geometry is a spherical capsid surrounded by a constellation of protruding peplomers. We take our bead-rod models of virus particles to be suspended in a Newtonian solvent. To any such collection of bead masses, we can associate a *moment of inertia ellipsoid* (MIE) whose center is the center of mass and whose principal moments of inertia match those of the virus particle. The MIE, thus, determines the orientability of the virus particle, and thus, the virus rotational diffusivity. We use Eqs. (3)–(13) in Ref. 1 for the method of computing the rotational diffusivity (see Footnote 2 of p. 62 of Ref. 10)

$$D_{r}\equiv \frac{1}{6\lambda },$$ (2)

or [Eq. (23) of Ref. 1]

$$\lambda_{0}D_{r}=\frac{\nu }{72},$$ (3)

which we will use for our results below. Symbols, dimensional, or nondimensional are defined in Table I or Table II, following the companion paper for singly beaded peplomer for SARS-CoV-2 particle.^1^

**TABLE II.** Dimensionless variables and groups.

Name	Symbol
Capsid-sphere	C
Coefficient in Eq. (3)	ν
Coefficient in Eq. (6)	a
Coefficient in Eq. (6)	b
Deborah number, oscillatory shear	De≡λω
Equilateral triangle of $i_{th}$ peplomer	$T_{i}$
Sphere	S
Total number of beads	N
Total number of capsid beads	$N_{c}$
Total number of peplomers	$N_{p}$
Weissenberg number	$\text{Wi}\equiv \lambda {\dot{\gamma }}^{0}$

**TABLE III.** Singly and triply beaded peplomer coronavirus particle characteristics from general rigid bead-rod theory.

Bulb beading	SARS-CoV-2	$\frac{I_{1}}{mL^{2}}$	$\frac{I_{2}}{mL^{2}}$	$\frac{I_{3}}{mL^{2}}$	a	b	ν	$\frac{2b}{a\nu }$	$\frac{\eta_{0}-\eta_{s}}{\mathrm{nkT}\lambda }$	$\frac{\lambda }{\lambda_{0}}$	$\lambda_{0}D_{r}$	$\frac{\Psi_{1,0}}{\lambda \left(\eta_{0}-\eta_{s}\right)}$
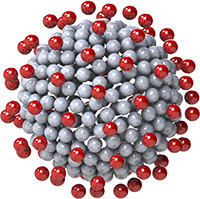	$N_{c}=256,\;N_{p}=74$	$2.48\times 10^{2}$	$2.48\times 10^{2}$	$2.48\times 10^{2}$	$1.24\times 10^{2}$	$1.19\times 10^{-8}$	$2.42\times 10^{-2}$	$7.96\times 10^{-9}$	$\frac{3}{2}$	$4.96\times 10^{2}$	$3.36\times 10^{-4}$	$1.60\times 10^{-8}$
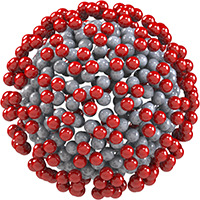	$N_{c}=256,$ $N_{p}=74$	$4.05\times 10^{2}$	$4.04\times 10^{2}$	$4.05\times 10^{2}$	$2.02\times 10^{2}$	$7.30\times 10^{-9}$	$1.50\times 10^{-2}$	$4.87\times 10^{-9}$	$\frac{3}{2}$	$8.09\times 10^{2}$	$2.06\times 10^{-4}$	$1.95\times 10^{-8}$

## III. OSCILLATORY SHEAR FLOW

In this paper, we focus on small-amplitude oscillatory shear flow (SAOS). For this flow field, for the molecular definition of *small amplitude*, general rigid bead-rod theory yields [Eq. (32) of Ref. 1]

$${\lambda \dot{\gamma }}^{0}\ll \frac{1}{\nu \sqrt{2}},$$ (4)

whose left side is the macromolecular Weissenberg number.

The polymer contributions to the complex viscosity,^11,12^

$$\eta^{*}\equiv \eta^{\prime }-{i\eta }^{\prime \prime },$$ (5)

are [Eqs. (40) and (41) of Ref. 8]

$$\frac{\eta^{\prime }-\eta_{s}}{\eta_{0}-\eta_{s}}={\left(\frac{1}{2b/a\nu }+1\right)}^{-1}\left(\frac{1}{2b/a\nu }+\frac{1}{1+{\left(\lambda \omega \right)}^{2}}\right),$$ (6)

and

$$\frac{\eta^{\prime \prime }}{\eta_{0}-\eta_{s}}={\left(\frac{1}{2b/a\nu }+1\right)}^{-1}\frac{\lambda \omega }{1+{\left(\lambda \omega \right)}^{2}},$$ (7)

where λω is the Deborah number. In this paper, we plot the real and *minus* the imaginary parts of the shear stress responses to small-amplitude oscillatory shear flow as functions of frequency, following Ferry (Secs. 2.A.4.-2.A.6. of Ref. 13) or Bird *et al.* (Sec. 4.4 of Ref. 14).

As ω→0, for the polymer contribution to the zero-shear viscosity, we get

$$\frac{\eta_{0}-\eta_{s}}{\mathrm{nkT}\lambda }=\frac{a\nu }{2}+b=b\left[1+\frac{2b}{a\nu }\right]{\left(\frac{2b}{a\nu }\right)}^{-1},$$ (8)

which we use in the table of Sec. V below.

## IV. MODELING OF TRIMERIC PEPLOMER

As shown by Kirchdoerfer,^15^ each trimeric peplomer head, consisting of three glycoproteins, is well-approximated by an equilateral triangle when viewed along the spike axis. In the general rigid bead-rod model,^1^ this trimer is replaced with a sphere. Here, we approximate the trimer by considering an identical point charge at each vertex of the equilateral triangle.

### A. Kinematics

Let $N_{p}$ be the number of trimeric peplomers attached to the capsid-sphere C of radius $r_{c}$. Let $T_{i}$ denote the equilateral triangle that approximates the trimeric head of the $i_{th}$ peplomer. Let the $p_{th}$ vertex of $T_{i}$ be parameterized by $r_{c}r_{i,q}$, where $i=1\ldots N_{p}$ and q=1,2,3. Let the length of the side of $T_{i}$, $i=1\ldots N_{p}$ be given by d. Thus, the vertices of $T_{i}$ are

$$r_{c}^{2}|r_{i,1}-r_{i,2}{|}^{2}=r_{c}^{2}|r_{i,1}-r_{i,3}{|}^{2}=r_{c}^{2}|r_{i,2}-r_{i,3}{|}^{2}=d^{2}.$$ (9)

Let ℓ be the length of the spike of each peplomer, with each spike normal to C at the point of contact on C. We assume that the centroid of $T_{i}$ is at the other end of the spike. Therefore, it must lie on the sphere S of radius $r_{s}=r_{c}+\ell$,

$$\frac{r_{c}^{2}{\left(r_{i,1}+r_{i,2}+r_{i,3}\right)}^{2}}{9}=r_{s}^{2},\;i=1\ldots N_{p}.$$ (10)

We also assume that each triangle $T_{i}$ lies in the tangential plane of the S at its centroid. This implies that normal to the plane of $T_{i}$ must align with the vector joining the centroid of $T_{i}$ to the center of S,

$$\left[\left(r_{i,1}-r_{i,2}\right)\times \left(r_{i,1}-r_{i,3}\right)\right]\cdot \left(r_{i,1}+r_{i,2}+r_{i,3}\right)=0,$$ (11)

which simplifies to

$$\left(r_{i,1}\times r_{i,2}\right)\cdot r_{i,3}+\left(r_{i,2}\times r_{i,3}\right)\cdot r_{i,1}+\left(r_{i,3}\times r_{i,1}\right)\cdot r_{i,2}=0.$$ (12)

### B. Energetics

Let each vertex of each triangle $T_{i}$, $i=1\ldots N_{p}$ be endowed with point charge Q. The total electrostatic energy of $N_{p}$ peplomers, constrained to the sphere S of radius $r_{c}$, is given by

$$E=\frac{Q}{4\pi \epsilon r_{c}}\sum_{i=1}^{N}\;\sum_{\begin{array}{c} j=1\\ j\neq i \end{array}}^{N}\;\sum_{q=1}^{3}\;\sum_{s=1}^{3}\;\frac{1}{\left|r_{i,q}-r_{j,s}\right|},$$ (13)

where *ϵ* is the dielectric permittivity. Using the constrained minimization approach of Ref. 16, we obtain numerical equilibrium solutions $r_{i,q}$, $i=1\ldots N_{p}$, and p=1,2,3 that locally minimize the energy in Eq. (13) while satisfying the kinematic constraints in Eqs. (9)–(12), for given values of $N_{p}$. Since the charge Q appears only as a prefactor in Eq. (13), its value plays no role in determining equilibrium solutions.

Our trimeric model amounts to a replacement for the Thompson problem,^5^ the objective of which is to find a state that distributes $N_{p}$ equilateral triads of charges over a unit sphere as evenly as possible, with minimum electrostatic energy. By contrast, Wales^6,7^ distributed $N_{p}$ single charges, providing solutions for a large set of values of $N_{p}$.

## V. RESULTS AND CONCLUSION

**FIG. 5.**
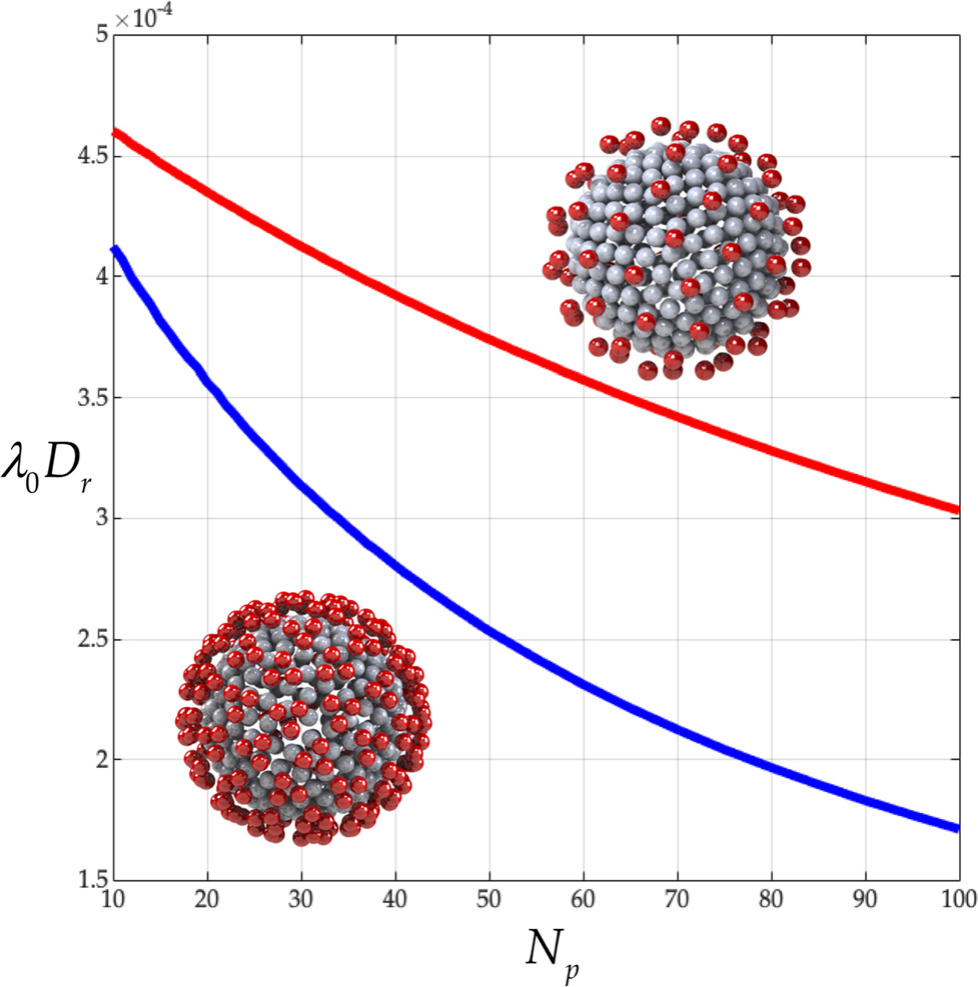
Dimensionless rotational diffusivity $\lambda_{0}D_{r}$ from Eq. (3) vs peplomer population $N_{p}$ ( $N_{c}=256$): single (red) and triple beading (blue) corresponds to, respectively, rows 1 and 2 of Table III.

From Fig. 5, we learn that the detailed triangular structure of the peplomer head and its singly beaded counterpart share the same qualitative behavior. For both, the rotational diffusivity, $\lambda_{0}D_{r}$, descends monotonically with $N_{p}$. However, the detailed triangular structure of the peplomer head reduces significantly $\lambda_{0}D_{r}$ of the coronavirus particle. Specifically, at the measured peplomer population of $N_{p}=74$, we see a reduction in $\lambda_{0}D_{r}$ of 39%. On close inspection, Fig. 5 also reveals

$$\frac{\lambda_{0}D_{r}\left(3N_{p}\right)}{\lambda_{0}D_{r}\left(N_{p}\right)}\approx 1,$$ (14)

that is, the dimensionless rotational diffusivity of a coronavirus with $N_{p}$ singly beaded peplomers has about the same dimensionless rotational diffusivity of a coronavirus with ${\frac{1}{3}N}_{p}$ triply beaded peplomers.

**FIG. 6.**
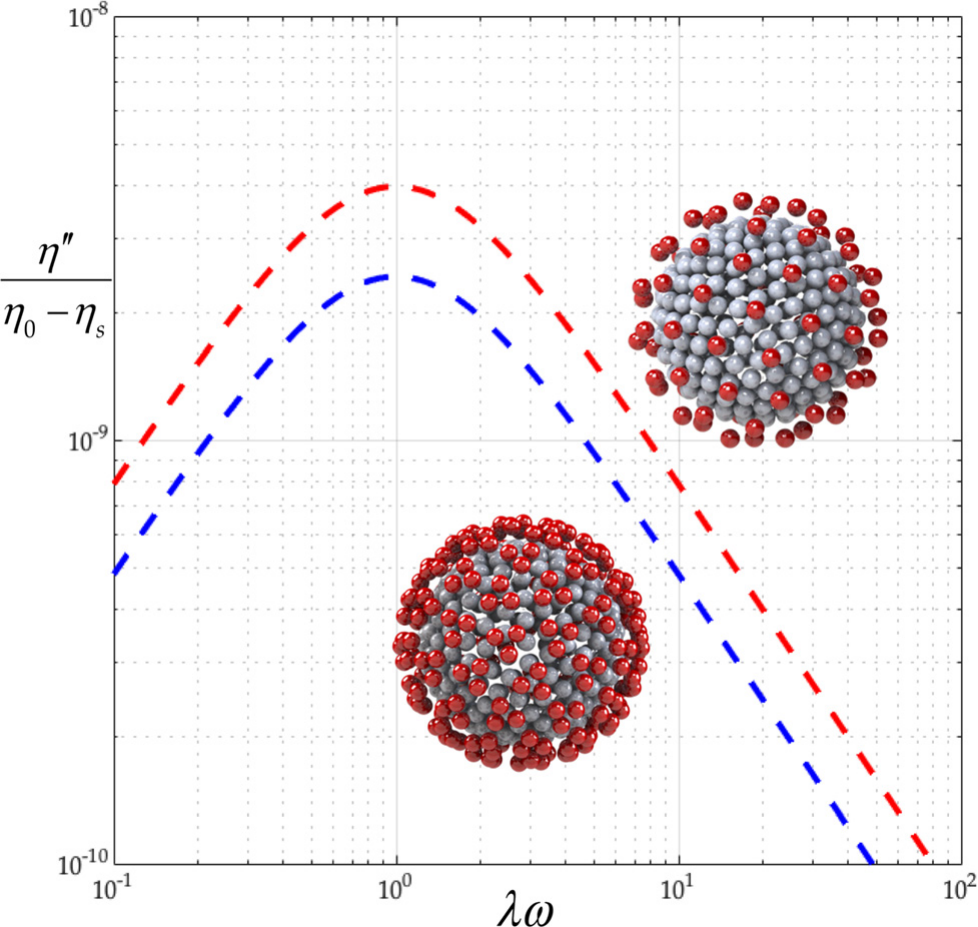
Effect of single (red) and triple beading (blue) on *minus* the imaginary part of the complex viscosity ( $N_{c}=256$, $N_{p}=74$ corresponds to, respectively, rows 1 and 2 of Table III). The polymer contribution $\left(\eta \prime -\eta_{s}\right)/\mathrm{nkT}\lambda$ is nearly constant: $\left(\eta_{0}-\eta_{s}\right)/\mathrm{nkT}\lambda =3/2$.

From Fig. 6, we learn that the elasticity, $\eta^{\prime \prime }/\left(\eta_{0}-\eta_{s}\right)$, of the coronavirus particle suspension is slight and that the detailed triangular structure of the peplomer head slightly reduces this elasticity. From Table III, we see that the corresponding b is nearly zero so that the polymer contribution to the real part of the complex viscosity is constant, $\left(\eta \prime -\eta_{s}\right)/\mathrm{nkT}\lambda =\left(\eta_{0}-\eta_{s}\right)/\mathrm{nkT}\lambda =3/2$. From Table III, we learn that the detailed triangular structure of the peplomer head increases the relaxation time, λ, and thus, decreases the zero-shear viscosity, $\eta_{0}$. From the rightmost column of Table III, we learn that the detailed triangular structure of the peplomer head decreases the zero-shear value of the first normal stress coefficient, $\Psi_{1,0}$.

Whereas much prior work on fluid physics related to the virus has attacked transmission,^17–41^ this paper focuses on the *ab initio* calculation of coronavirus transport properties. Specifically, we have determined the rotational diffusivity, the property governing the particle alignment for cell attachment (see Sec. I of Ref. 1). Although our work is mainly curiosity driven, it may deepen our understanding of drug, vaccine, and cellular infection mechanisms.

Chaurasia *et al.*^42^ (see also Chaurasia^43^) developed a framework to find equilibrium solutions of a system consisting of flexible structures, specifically charged elastic loops constrained to a sphere. Their framework could be used to model flexible peplomers with uniformly charged heads. We leave this daunting task for a future study.

Since the coronavirus capsid can be ellipsoidal (Fig. 3. of Ref. 44), called *pleomorphism*, we must eventually consider this too. Whereas this work considered the detailed triangular structure of the peplomer head as triads of three point-charges, we could also consider uniformly charged triangular rigid peplomers constrained to a sphere. By *uniformly charged triangular*, we mean that the charge would be uniformly distributed over the edges of the triangle rather than point charges at its vertices. We leave this task for a later date.

One cognate transport problem is the transient translation and twist of coronavirus spikes rearranging freely under their own electrostatic repulsions, for instance, the transient following the extraction of a single spike. This paper is, of course, silent on this interesting problem, which we leave for another day.

As in our previous work,^1^ we have used repulsions of charged particles over the surfaces of spheres for both the capsid and the peplomer heads of the coronavirus to arrive at its transport properties. It has not escaped our attention that our solutions to the Thomson problem can also be used to calculate the Young's modulus of the coronavirus particle [Eq. (3a) of Ref. 45] and that by extension this Young's modulus will depend upon peplomer population. We leave this calculation for another day. When using the references cited herein, it is best to be mindful of corresponding ganged errata in Ref. 46.

## Data Availability

The data that support the findings of this study are available within the article.
